# Comparing Analgesic Efficacy: Ropivacaine with Dexamethasone vs. Ropivacaine with Dexmedetomidine After Cesarean Section Using Transversus Abdominis Plane Block (2020 - 2021)

**DOI:** 10.5812/aapm-147872

**Published:** 2024-11-18

**Authors:** Mohammadreza Jamshidi, Mona Ghaderi, Mitra Hojatansari

**Affiliations:** 1 School of Medicine, Zanjan Medical University, Zanjan, Iran; 2 Department of Emergency and Critical Care Nursing, School of Nursing and Midwifery, Zanjan University Sciences, Zanjan, Iran

**Keywords:** Cesarean Section, TAP Block, Ropivacaine, Dexmedetomidine, Dexamethasone

## Abstract

**Background:**

There are several methods for managing postpartum pain. The combined use of drugs with anesthetics can lead to effective pain management.

**Objectives:**

The present study aimed to compare the analgesic effects of ropivacaine (RPV) + dexamethasone (DEXA) and RPV + dexmedetomidine (DEX) on pain after cesarean section (CS) using the transversus abdominis plane (TAP) block.

**Methods:**

This double-blind, randomized clinical trial employed a quadruple block randomization method and included 40 participants scheduled for CS at Ayatollah Mousavi Hospital in Zanjan, Iran, during 2020 - 2021. The participants were divided into two groups: The first group received 15 mL of RPV 2% combined with 100 µg of DEX via the bilateral TAP block method, while the second group received 15 ml of RPV 2% combined with 8 mg of DEXA. The analgesic effects of the two drug combinations were evaluated at 0, 3-, 6-, 12-, and 24-hours post-CS using the visual analog scale (VAS) to measure pain intensity. Data analysis was conducted using SPSS software, version 24.

**Results:**

In the RPV + DEX group, the onset of pain was delayed, resulting in a longer duration before the administration of painkillers (P = 0.041 and P < 0.001). However, pain intensity between 3- and 24-hours post-surgery was significantly higher in the RPV + DEX group compared to the RPV + DEXA group (P = 0.028, P < 0.001). The RPV + DEX group experienced longer durations before the onset of pain and the need for painkillers (P = 0.041, P < 0.001). Hypotension was more frequently observed in the RPV + DEXA group at 0 hours (P = 0.068) and 3 hours post-surgery (P = 0.003). Additionally, bradycardia and sedation incidences were higher in the RPV + DEXA group at 3 hours post-surgery (P = 0.005, P = 0.048).

**Conclusions:**

The use of RPV + DEXA, unlike RPV + DEX, demonstrated positive and significant effects on pain management in female CS candidates using the TAP block method, despite its side effects.

## 1. Background

Cesarean section (CS) is a prevalent procedure in obstetrics that involves delivering a fetus through an incision in the abdominal wall and uterus (1). This surgery often leads to moderate to severe pain within the first 48 hours post-operation, impacting breastfeeding and mother-infant interaction (2). The transverse abdominis plane (TAP) block is an anesthetic technique in which local anesthetic (LA) drugs are injected into the TAP, located between the internal oblique (IO) and transversus abdominis (TA) muscles (3). This method anesthetizes the abdominal wall nerves, thereby reducing the need for narcotics and decreasing postoperative nausea and sedation, although it offers only short-duration analgesia (4, 5).

Recent studies have reported that using the TAP block with ropivacaine (RPV), a LA, can significantly reduce opioid consumption following CS (6-9). Ropivacaine is typically employed for local, regional, or epidural anesthesia in the lumbar or sacral regions. Its mechanism of action involves reducing the permeability of nerve cells to sodium, thereby increasing the action potential threshold and preventing the generation and conduction of nerve signals (10). Ropivacaine is preferred over short-acting drugs such as mepivacaine or lidocaine due to its efficacy in prolonging postoperative analgesia and extending the duration of nerve blocks (11).

Despite the benefits of long-term nerve blocks, particularly post-surgery, increasing the concentration or dosage of RPV to prolong analgesia can lead to local and systemic toxicity. To extend the duration of analgesia after CS without increasing the drug concentration or dose, and to minimize side effects, including motor neuron block, various adjunctive drugs have been investigated to enhance the therapeutic effects of LAs (12).

Dexmedetomidine (DEX) is an alpha-2 adrenergic receptor agonist known to lower the shivering threshold. It exhibits sedative, analgesic, sympatholytic, and hemodynamic modulation effects while also reducing anesthetic requirements. Adding DEX to RPV at 0.5% has proven more effective than fentanyl for diabetic patients undergoing surgery (13, 14). Recent findings indicate that DEX can reduce the incidence of postoperative shivering (15).

Dexamethasone is a long-acting glucocorticoid with an anti-inflammatory potency 25 times greater than that of cortisol, and it lacks mineralocorticoid properties (16). Glucocorticoids can alleviate peripheral pain by altering plasma endorphin levels (17). Numerous studies highlight the effectiveness of drug combinations in reducing postoperative pain following CS (18-21). However, research has shown that RPV alone is insufficient for pain control after CS (22, 23). Literature reviews reveal that combining DEXA with RPV in the TAP block technique can extend analgesia duration in various abdominal surgeries (16, 24, 25).

A study by Gao et al. suggests that dexamethasone does not extend the duration of sensory block as effectively as DEX. This finding highlights the need for further research with varying dosages to determine the optimal dose of dexamethasone for enhancing the sensory block induced by anesthetic agents. To address this knowledge gap, we designed the current study (26). To date, no study has evaluated the combined effect of RPV with both DEX and DEXA in CS. Therefore, we aimed to compare the analgesic effects of RPV + DEXA and RPV + DEX on postoperative pain following CS using the TAP block method.

## 3. Methods

### 3.1. Study Design

**Figure 1.**
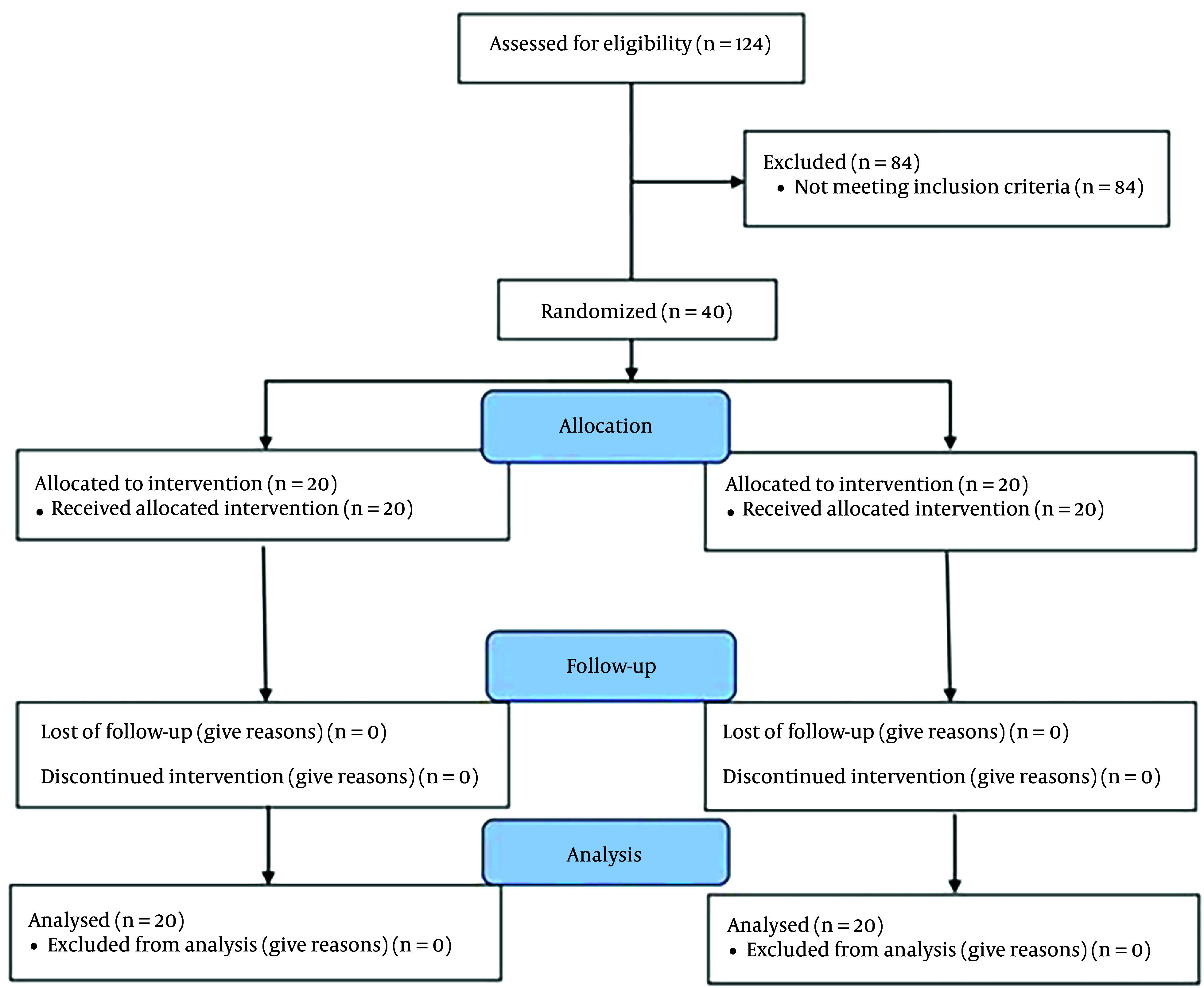
The entry and exit of patients in the present study based on Consort criteria.

This randomized controlled trial was conducted from April 2021 to December 2021, utilizing a randomized block design method. The inclusion criteria included patients classified as American Society of Anesthesiologists (ASA) class I - II, candidates for elective CS, suitable for spinal anesthesia, and with a Body Mass Index (BMI) of less than 30. Exclusion criteria were elective surgeries lasting more than three hours, contraindications to spinal block (such as coagulopathy, neurological disorders, spinal infection, or allergies to LAs), visible spinal deformities, the potential need to switch to general anesthesia, a history of chronic or acute headaches, a BMI over 30, recent spinal anesthesia or epidural within the past 15 days, drug addiction, and drug allergies. Additionally, subjects who experienced mortality during the study period, patients with bedsores, those using psychiatric medications, or those who had previously used painkillers before undergoing CS were excluded from the study (Figure 1). 

The study protocol was approved by the Ethics Committee of Zanjan University of Medical Sciences, Zanjan, Iran, under the ethical code IR.ZUMS.REC.1400.022. Written informed consent was obtained from all participants.

### 3.2. Intervention Groups 

Using a non-probability sampling method, 40 women scheduled for non-emergency CS and referred to Ayatollah Mousavi Hospital, part of Zanjan University of Medical Sciences (Iran), were selected for this study. Patients aged 18 - 35 years were randomly assigned to two groups of 20 each. Based on the number of groups and the calculated sample size, six permutations (BAAB, BABA, BBAA, ABAB, AABB, and ABBA) were considered. These permutations were written on six cards, which were then selected randomly to determine the order of group assignment.

The first group received 15 mL of RPV 2% plus 100 µg of DEX using the bilateral TAP block method, while the second group received 15 mL of RPV 2% plus 8 mg of DEXA. Both RPV and DEX were provided by VARIAN Farmed, Iran (17). All blocks were administered by a single specialist (the first author), which precluded blinding during the procedure; thus, patients were informed about the injected drug. However, the resident who recorded the results (the second author) was unaware of the group assignments, ensuring that the study was conducted in a blinded manner.

Based on findings from a comparable study (17), it is projected that supplementing RPV with DEX could delay pain onset by approximately 1.1 hours, with a standard deviation of 1.22 hours. Using Power and Sample Size software and accounting for a 0.05 probability of type I error and 80% power, the required sample size was calculated to be 18 individuals per group. To account for a 10% attrition rate, the final sample size was adjusted to 20 individuals per group, totaling 40 participants.

All participants received spinal anesthesia via the classic paramedian method, performed by an anesthesiologist. This technique involves identifying the L3-L4 or L4-L5 intervertebral space, then moving the insertion point 1 cm laterally and 1 cm downward. The needle is angled 10 - 15 degrees medially and 10 - 15 degrees towards the pelvis to reach the subarachnoid space, where 15 mg of 0.5% bupivacaine (VARIAN Farmed, Iran) is injected.

The ultrasound-guided technique is considered the gold standard for TAP blocks due to its ease of use and safety, allowing for direct visualization of the needle before injecting the LA. Patients are positioned supine during the procedure. A high-frequency linear or curved ultrasound transducer with gel is placed on the abdomen for optimal contact and ultrasound wave transmission. In ultrasound imaging, the skin and subcutaneous fat appear as the most superficial layers, with three muscle layers beneath them: External oblique, IO, and TA. The IO muscle is typically the thickest, while the TA muscle is the thinnest. If the layer boundaries are unclear, adjusting the ultrasound depth can confirm the presence of the bowel below the TA muscle. Posterior scanning shows the IO and TA muscles meeting to form the thoracolumbar fascia. Internally, the aponeuroses of these muscle layers converge to form the rectus sheath.

Once the TAP compartment is identified with the ultrasound probe, the skin is infiltrated with lidocaine, and a block needle is inserted using the in-plane technique while ensuring continuous visualization of the needle tip with ultrasound. The needle is advanced between the IO and TA muscles, and LA is slowly injected after confirming negative aspiration of blood. As the LA is injected, the TAP compartment separates, hydrodissects, or "unzips," pushing the TA muscle downward. We administered 15 mL of the solution on each side of the patient. 

After the injection, patients were taken to the recovery room and monitored for vital signs, postoperative pain, pulse oximetry, and side effects. For patients with a pain score higher than 4 on the Visual Analog Scale (VAS), oral acetaminophen tablets were prescribed. Pain levels were monitored for 24 hours post-surgery. 

In this study, we developed a research instrument that included demographic information, a pain assessment tool, and information on drug side effects. Pain intensity was measured using the VAS, a validated and reliable measure that is widely used in numerous studies. The checklist was completed by the second author, who is the resident responsible for the thesis.

The patient's baseline blood pressure was recorded before the intervention, with a drop exceeding 20% considered hypotension, which was initially managed with fluid therapy. If hypotension persisted despite fluid therapy, 5 mg of vasopressor medication was administered intravenously. Heart rate was also assessed before the intervention, and bradycardia (defined as a heart rate below 55 beats per minute) was treated with 0.6 mg of intravenous atropine. Pain management was stratified based on pain intensity: Oral acetaminophen for pain scores between 3 and 5, additional painkillers for scores above 5, and narcotic drugs (pethidine) intravenously for scores exceeding 7. Inadvertent intravascular injection of DEX during the procedure resulted in patient sedation; if observed, patients were monitored until full consciousness was regained. Subsequently, all patients underwent monitoring, with blood pressure and heart rate assessed using oximetry before and after cesarean delivery. Drug side effects following CS included apnea, hypotension, prolonged unconsciousness, nausea, and vomiting at 3-, 6-, and 12-hours post-surgery, as well as 24 days after the procedure.

### 3.3. Instruments 

Data collection utilized a checklist encompassing demographic information such as age, gender, weight, and medical history. The checklist underwent validation by five experts in the Department of Pediatrics at Zanjan University of Medical Sciences. Pain intensity was evaluated using the VAS, a self-report tool endorsed in Iran, comprising a 10-cm line ranging from zero (no pain) to ten (severe pain). Scores of 1 - 3, 4 - 6, and 7 - 10 corresponded to mild, moderate, and severe pain, respectively.

### 3.4. Data Analysis 

The collected data were entered into SPSS software (version 24, IBM, Armonk, NY) and summarized using the mean (standard deviation) for continuous variables and frequency (percentage) for categorical variables. Inter-group comparisons were performed using the Independent *t*-test, Mann-Whitney test, chi-square test, and logistic regression model as appropriate. Statistical significance was determined at a threshold of P < 0.05.

## 4. Results 

Throughout the study period, 124 individuals underwent evaluation for CS, out of which 40 met the inclusion and exclusion criteria and were included in our study. The study commenced and concluded with 40 participants (twenty in each group) (Figure 1). 

In both the RPV + DEX and RPV + DEXA groups, the mean (SD) age of participants was 25.3 (31.3) years and 27.0 (1.81) years, respectively, showing no statistically significant difference (P = 0.134). Similarly, the mean (SD) gestational age of mothers in the RPV + DEX and RPV + DEXA groups was 34.60 (1.27) weeks and 35.50 (1.93) weeks, respectively, with no significant difference noted (P = 0.090). However, it is noteworthy that the BMI of mothers in the RPV + DEX group was significantly higher than that in the RPV + DEXA group (P = 0.018). Regarding the ASA classification, there was no significant difference between the mothers in the RPV + DEX group and those in the RPV + DEXA group (P = 0.723). This indicates that both groups had similar overall health statuses in terms of anesthesia risk stratification. Table 1 provides a comprehensive overview of these demographic and clinical characteristics, allowing for a more detailed examination of the differences between the two groups.

**Table 1.** Comparison of Demographic and Basic Variables Between the Two Groups Participating in the Study ^a^

Variable and Category	Ropivacaine Dexmedetomidine Group (n = 20)	Ropivacaine Dexamethasone Group (n = 20)	P-Value
**Age, (y)**	25.30 (3.31)	27 (1.81)	0.134 ^b^
**Gestational age, (w)**	34.60 (1.27)	35.50 (1.93)	0.090 ^c^
**BMI, kg/m** ^**2**^	27.55 (3.18)	25.40 (3.27)	0.018 ^c^
**ASA**			0.723 ^d^
I	14 (70)	15 (70)	
II	6 (30)	5 (25)	

^a^ Values are expressed as mean (SD).

^b^ Independent *t*-test.

^c^ Mann-Whitney test.

^d^ Chi-square test.

Moving on to the assessment of postoperative pain intensity following CS, Table 2 presents the mean (SD) pain intensity at various time points, including 0, 3, 6, 12, and 24 hours postoperatively. Notably, the pain intensity between 3- and 24-hours post-operation was significantly higher in the RPV + DEX group compared to the RPV + DEXA group (P = 0.028, P < 0.001, respectively). 

**Table 2.** Comparison of Pain Measured at Different Times Between the Two Groups Participating in the Study ^a^

Variable and Time	Ropivacaine Dexmedetomidine Group (n = 20)	Ropivacaine Dexamethasone Group (n = 20)	P-Value Unjustified	P-Value Justified ^b^
**Pain intensity ** ^**c**^				
0	1.3 (0.47)	1.20 (0.41)	0.602	0.560
3	2.35 (0.49)	1.8 (0.77)	0.033	0.028
6	2.7 (0.73)	2.5 (0.76)	0.035	0.024
12	3.65 (0.67)	2.5 (0.76)	< 0.001	< 0.001
24	3.75 (0.97)	2.5 (0.51)	< 0.001	< 0.001

^a^ Values are expressed as mean (SD).

^b^ Adjusted for Body Mass Index.

^c^ Adjustment by Benjamini-Hochberg method using an independent *t*-test.

This indicates that individuals in the RPV + DEX group experienced more pronounced pain between 3 and 24 hours postoperatively than those in the RPV + DEXA group.

Table 3 further elaborates on the duration between surgery completion and pain onset, as well as the duration of painkiller requirement. Notably, both these durations were significantly longer in the RPV + DEX group compared to the RPV + DEXA group (P = 0.041 and P < 0.001, respectively). This suggests that participants in the RPV + DEX group experienced a delayed onset of pain, resulting in a longer interval before pain relief intervention was needed.

**Table 3.** Comparison of the Duration Between the Completion of Surgery and the Onset of Pain, as well as the Onset of the Need for Medication Between the Two Groups Participating in the Study ^a^

Variables	Ropivacaine Dexmedetomidine Group (n = 20)	Ropivacaine Dexamethasone Group (n = 20)	P-Value Unjustified	P-Value Justified ^b^
**The time between the end of surgery and the onset of pain**	7.95 (1.23)	5.7 (1. 34)	0.028	0.041
**The duration of the need for painkillers, hour**	6.2 (1.15)	5.45 (0.60)	< 0.001	< 0.001

^a^ Values are expressed as mean (SD).

^b^ Adjusted for body mass index using an independent *t*-test.

In terms of complications post-CS, including hypotension, bradycardia, sedation, nausea, and vomiting, no significant differences were observed between the two groups at 0-, 6-, 12-, and 24-hours post-surgery. However, it is noteworthy that hypotension at 0 hours was observed in two patients in the RPV + DEX group and six patients in the RPV + DEXA group, with no significant difference noted between the groups at this time point (P = 0.068).

Of the two patients who experienced hypotension in the RPV + DEX group, only one required vasopressor administration (5 mg intravenous ephedrine), while the others were managed with fluid therapy (normal saline). Conversely, at 3 hours post-surgery, hypotension was observed in two patients in the RPV + DEXA group who received fluid therapy, while no cases of hypotension were detected among RPV + DEX group patients. This difference between the two study groups was statistically significant (P = 0.003).

At 0 hours post-CS, bradycardia, sedation, nausea, and vomiting were observed as complications in only one patient from the RPV + DEX group. In contrast, bradycardia and sedation were noted in three cases, while nausea and vomiting were noted in four cases in the RPV + DEXA group. However, this difference was not statistically significant at 0 hours. Similarly, at 3 hours post-surgery, there was no significant difference between the two groups in terms of nausea and vomiting (P = 0.098). Nonetheless, a statistically significant difference was observed between the two groups at 3 hours for bradycardia and sedation, which were more prevalent in the RPV + DEXA group (P = 0.005 and P = 0.048, respectively). 

Table 4 provides a detailed breakdown of these complications, facilitating a comprehensive comparison between the two study groups.

**Table 4.** Comparison of Complications After Intervention at Different Time Points Measured Between the Two Groups Participating in the Study

Variable and Time	Ropivacaine Dexmedetomidine Group (n = 20)	Ropivacaine Dexamethasone Group (n = 20)	P-Value Unjustified	P-Value Justified ^b^
**Hypotension**				
0	2 (1)	6 (4)	0.114	0.068
3	-	2 (10)	0.487	0.003
**Bradycardia**				
0	1 (5)	3 (15)	0.292	0.262
3	-	1 (5)	> 0.999	0.005
**Sedation**				
0	1 (5)	4 (20)	0.342	0.15
3	1 (5)	4 (20)	> 0.999	0.098
**Nausea and vomiting**				
0	1 (5)	4 (20)	0.342	0.15
3	1 (5)	2 (10)	> 0.999	0.098

^a^ Values are expressed as mean (SD).

^b^ Logistic regression model (adjusted for body mass index).

## 5. Discussion 

The present study compared the analgesic effect of RPV + DEXA with that of RPV + DEX on pain in pregnant women undergoing CS using the TAP block method. The results indicated that the addition of DEX, compared to DEXA, to RPV using the TAP block method resulted in a significant difference in pain control. Additionally, a statistically significant difference was observed between the two study groups regarding the time to return of pain in patients. Consequently, the pain intensity (except at 0 hours), the duration of analgesia, and the need for painkillers to manage pain in the group receiving DEXA were significantly lower than in the group receiving DEX. Regarding complications, hypotension, bradycardia, and sedation were significantly lower in the group receiving DEX (only at 3 hours) than in the group receiving DEXA, while at other time points, the differences were nonsignificant. Furthermore, there was no significant difference in nausea and vomiting between the two study groups at 0, 3, 6, 12, and 24 hours after CS.

The majority (80%) of patients undergoing surgery reported experiencing postoperative pain. Inadequate postoperative analgesia can have detrimental effects on pulmonary function and may also increase sensitivity to painful stimuli, potentially leading to chronic pain syndrome (15). Effective postoperative pain management is crucial not only for hemostasis but also for reducing treatment costs and shortening the length of a patient's recovery and hospitalization. The use of analgesics, such as opioids, alpha-2 agonists, neostigmine, and vasoconstrictors, in combination with local anesthesia, is beneficial for enhancing analgesia and minimizing anesthesia-related complications (16). Clonidine and DEX exert their effects through presynaptic and postsynaptic alpha-2 receptors (27). 

Consistent with our findings, the study by Singla et al. demonstrated that combining DEXA with RPV for TAP block after CS resulted in reduced postoperative pain. Furthermore, DEXA provided superior pain relief compared to fentanyl (17). 

In line with the findings of our study, recent research by Stephan et al., Zhu and Sun, and Singh et al. indicated that incorporating 8 mg of DEXA into peripheral local anesthetic injections prolonged the duration of peripheral nerve block analgesia, optimizing efficacy (28-30). Similarly, our study's results, akin to those of Yi-Han et al. and Sinha et al., showed that augmenting RPV with DEXA significantly extended the analgesic effect of RPV post-surgery, aligning with previous research outcomes. The adjunctive use of DEXA with RPV is believed to prolong the duration of analgesia, enhance variability in analgesic duration, and markedly reduce the need for postoperative narcotics. Multiple studies have affirmed the safety of DEXA as an adjunct to RPV anesthesia solution (31, 32).

Research has suggested that adding DEX to RPV in epidural and caudal anesthesia produces long-lasting postoperative analgesia with minimal side effects (17, 33, 34). Supplementing RPV with DEX significantly prolongs the duration of sensory and motor block by improving the quality of analgesia after surgery compared to RPV alone (35). According to recent studies by Yang et al., Pan et al., Li et al., and Yang et al., and in line with our findings, both DEXA and DEX are effective for pain management in women undergoing CS when administered via the TAP block technique. Nonetheless, DEXA has demonstrated superior efficacy compared to DEX (16, 23-25).

### 5.1. Limitations and Recommendations 

Our study was conducted at a single center, and the sample size was relatively small. Additionally, we did not assess stress levels, depression, sleep quality, or pain tolerance in our patients, despite their known influence on postoperative pain severity. Therefore, future studies should address these limitations to enhance the generalizability of the findings.

### 5.2. Conclusions

Our results indicated that the addition of DEXA to RPV, compared to DEX with RPV, in the TAP block method for women undergoing CS led to a decrease in pain intensity, an increase in the duration of analgesia, and a reduction in the need for opioid administration. Considering the differences in the effects of these two drugs, it is recommended to use a combination of RPV with DEXA in TAP block for women who are candidates for CS to effectively manage this group.

## Data Availability

The dataset presented in the study is available upon request from the corresponding author during submission or after publication. The data are not publicly available due to the confidentiality of patient information.

## Clinical Trial Registration Code:

IRCT20210302050553N1.
